# Assessment of Physicochemical Properties of Dust from Crushing High-Carbon Ferrochrome: Methods for Agglomeration

**DOI:** 10.3390/ma18040903

**Published:** 2025-02-19

**Authors:** Otegen Sariyev, Assylbek Abdirashit, Maral Almagambetov, Nurzhan Nurgali, Bauyrzhan Kelamanov, Dauren Yessengaliyev, Azamat Mukhambetkaliyev

**Affiliations:** 1 Department of Metallurgy and Mining, K. Zhubanov Aktobe Regional University, Aktobe 030000, Kazakhstan; samgauaktobe@gmail.com (O.S.); kelamanovb84@gmail.com (B.K.); 6d070900dauren@gmail.com (D.Y.); zika8529@gmail.com (A.M.); 2 ERG Research and Engineering Center, Astana 010000, Kazakhstan; maral.almagambetov@erg.kz (M.A.); nurzhan.nurgali@erg.kz (N.N.)

**Keywords:** recycling, aspiration dust, HC FeCr, briquette, smelting, agglomeration

## Abstract

Fine classes of metal dust generated during the production of ferroalloys increase the likelihood of irretrievable losses, creating the prerequisites for the development of rational methods for processing this material. One of the known technologies for recycling dispersed raw materials in metallurgical processing is their direct remelting. Although this technology is easily feasible, it has several significant drawbacks, among which the main problem remains the high dust carryover of fine material by ascending gas-thermal flows. A potential solution could be the preliminary preparation of raw materials through agglomeration. Domestic enterprises producing various types of ferroalloys have the necessary infrastructure and equipment for agglomerating dispersed ore materials, but the lack of experience and resource-saving technologies for processing metal dust prevents their full integration into metallurgical processing. In this regard, there is significant interest and demand from ferroalloy enterprises for the development of new methods to involve dispersed metal production waste in secondary recycling, adapted to existing agglomeration equipment. Numerous studies have shown that the cheapest method of agglomeration is briquetting. Given the advancement of briquetting technologies, as well as the use of the latest equipment and binding materials in this process, it can be assumed that this will allow for more complete integration of aspiration dust from ferrochrome crushing into metallurgical processing. To test this assumption, studies were conducted on the physicochemical properties of aspiration dust from ferrochrome crushing, assessing the possibility of obtaining an agglomerated product with the required strength parameters. The results of these studies demonstrated the fundamental possibility of producing high-carbon ferrochrome from briquetted material made from aspiration dust of ferrochrome crushing.

## 1. Introduction

The production of ferroalloys is accompanied by the formation of by-products, such as slags, dust from raw and charge materials, and commodity metal during handling, crushing, and fractionation. Slags generated from high-carbon ferrochrome (HC FeCr) production are entirely processed into crushed stone, and as such, there are currently no issues with their utilization. Dust from charge materials collected from aspiration and gas cleaning systems of furnace units is recycled by agglomeration and returned to the main production cycle. However, the issue of efficiently utilizing aspiration dust from HC FeCr crushing (AD) remains unresolved [1]. The question of the efficient utilization of aspiration dust from the crushing of high-carbon ferrochrome (AD) remains open. The annual volume of this dust generated at two ferroalloy plants in Aktobe and Aksu, taking into account the commissioning of new facilities in 2014, amounts to approximately 5000 tons [2,3].

AD dust is highly dispersed, and its recycling through remelting in bulk is associated with significant mechanical losses during furnace loading, burn-off, and dust carryover into the gas cleaning system [4,5,6]. Preliminary data indicate that losses can reach up to 15% of the annual AD generation volume. This highlights the need to minimize or eliminate such losses through rational methods of recycling. One well-known approach to utilizing dispersed materials is agglomeration [7,8,9,10,11,12,13,14] or SHS (Self-Propagating High-Temperature Synthesis) technologies [15,16]. Certain types of ferroalloy production dust are used as components of sintering charges [17], raw materials for refractory production [18], and low-carbon ferrochrome production [19]. In studies [20,21], aspiration dust briquetted with steel shavings and coke was used to produce ferrosilicochrome. Additionally, in works [22], ferrochrome powder was utilized as a binder material, partially replacing Portland cement.

To determine the most rational methods for recycling the aforementioned material (AD), it is essential to first study its physico-chemical properties. Obtaining more comprehensive information of this nature will facilitate the selection of the most simple, yet economically viable technology for incorporating AD into secondary processing.

Considering the experience of domestic enterprises in processing dry gas-cleaning dust from ferroalloy furnaces and the lack of industrial experience in processing metal dust, this research primarily focuses on the agglomeration of aspiration dust (AD) formed during the crushing of high-carbon ferrochrome. The briquetting method was chosen as the base technology for processing due to its accessibility and economic feasibility as a way to dispose of dispersed materials.

Unlike traditional approaches based on direct melting or agglomeration using standard binders, this work proposes the use of dust from baghouse filters in dry gas cleaning systems (PSG) as a filler in the briquetting of AD. Research into the physicochemical properties of AD and PSG substantiated their joint use, enhancing the strength properties of the briquettes by improving interparticle adhesion and heat resistance of the agglomerated material.

Laboratory tests on the briquetting technology were conducted to adapt existing briquetting equipment in enterprises to new polymer-based binders, replacing traditional sodium liquid glass, cement, and bentonite. The use of the modified mixture composition allowed the production of briquettes that meet mechanical and thermal strength requirements and confirmed the possibility of their metallurgical processing to produce conditioned ferrochrome.

## 2. Materials and Methods

The most important criteria for the physico-chemical properties of materials are their chemical composition and particle size distribution. Data on these parameters for AD are presented below in Table 1 and Table 2. The chemical composition was determined in an accredited chemical laboratory using titrimetric analysis (Table 1).

The chemical composition of AD, in terms of its main elements and impurities, is nearly identical to that of standard HC FeCr grades (Cr: 65–68%, Si: 1–2%, and C: 6–9.5%). This similarity should not result in any difficulties or deviations (regarding the primary element content and impurities) during secondary metallurgical processing. To determine the granulometric composition of the aspiration dust (AD), a sieve analyzer model ASV-200 was used, which is applied for dry sieving.

The analysis of the particle size distribution of AD shows that the main size class is represented by the fraction below 0.071 mm. Typically, such fine fractions in ferrous metallurgy undergo granulation and high-temperature sintering. In our case, high-temperature sintering is not acceptable due to the high risk of oxidation of the primary element.

Another important factor influencing the selection of a recycling technology for dispersed materials is the phase composition, structure, and grain shape.

Studies using X-ray diffraction analysis (XRD) on a DRON-2 setup revealed that the phase composition of the aspiration dust (AD) is primarily represented by chromium carbide (Cr_7_C_3_), with a minor presence of free graphite.

**Figure 1:**
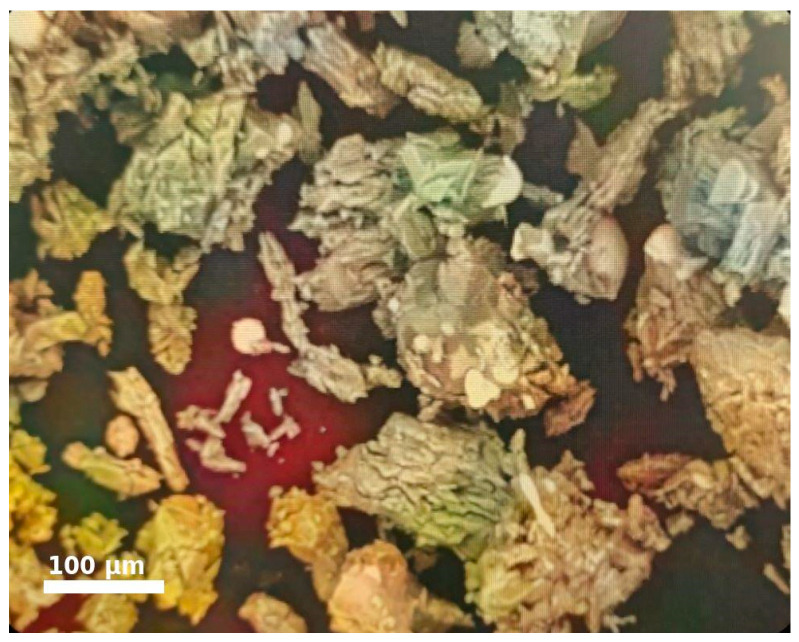
Grain Shape of AD under Microscopic Examination.

During metallographic examination under a microscope model MIM-8M, the particles (grains) of the aspiration dust (AD) exhibited irregular, sharp shapes, and needle-like crystals were also observed (Figure 1). The AP samples were placed in plastic sleeves, embedded in epoxy resin, and polished using diamond abrasive materials on an MP-1S metallographic grinding and polishing machine. The studies were conducted using a metallographic microscope model MIM-8M.

Such particle shapes cause certain difficulties during agglomeration, related to the variable surface area of contact between the base material and binder. The low contact area, in turn, leads to the formation of voids between the agglomerated particles, which require an increased binder consumption to fill [3].

One of the known methods in such cases is the use of fillers that are more dispersed in their granulometric composition. Fillers help fill the voids between the particles and enhance their adhesion by increasing the contact area between the material and the binder. This, in turn, leads to savings in binder usage and results in stronger agglomerated materials.

Considering that AD is essentially a dispersed form of HC FeCr, it is reasonable to consider filler materials that contain chromium oxides to minimize the risks of diluting the primary element. From this perspective, dust from dry gas cleaning systems of ferroalloy furnaces, particularly from HC FeCr production, could be of interest. The data on the average chemical composition of dust, determined using titrimetric analysis, can be found in Table 3.

**Figure 2:**
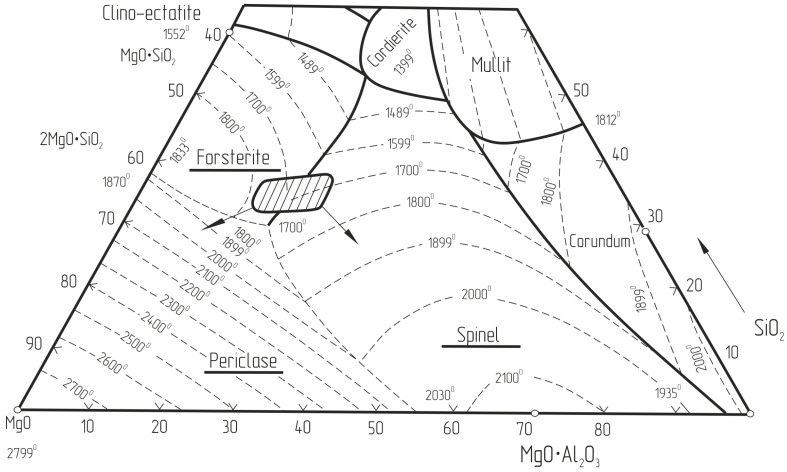
Region of the Dust Composition (shaded) on the MgO-SiO_2_-Al_2_O_3_ Phase Diagram.

From the data in Table 3, it is evident that the investigated materials represent a magnesia-silicate-spinel system. When recalculating this composition to the three main oxides of the MgO-SiO_2_-R_2_O_3_ system (R = Al, Cr, Fe), it is possible to determine the existence region on the ternary phase diagram (Figure 2).

The granulometric composition of the dust is predominantly represented by the particle size class of minus 0.02 mm, accounting for more than 87% (Table 4). The granulometric analysis was performed using the “wet” sieving method with an Analysette 3 [Fritsch, Germany Year of production: 2014] Spartan vibratory sieve shaker.

**Figure 3:**
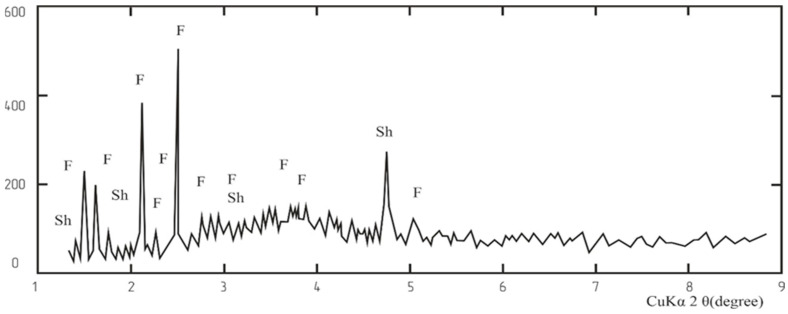
XRD Diagram of the Dry Gas Cleaning Dust Sample.

The X-ray diffraction (XRD) analysis of the dust phase composition revealed the presence of forsterite, spinelid, and a glass phase of variable composition (main phases), along with a small amount of magnesium aluminosilicates (impurity phases) (Figure 3).

**Figure 4:**
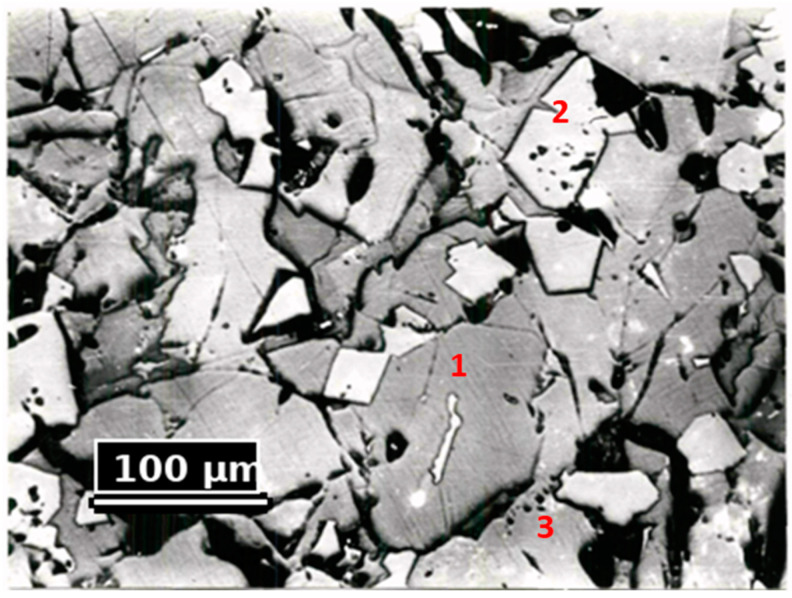
Microstructure of the Studied Dust Sample (Magnification 100×, Light). 1—Forsterite, 2—Spinelid, and 3—Glass.

Petrographic studies were conducted to refine the material composition. The dust samples were placed in aluminum sleeves, filled with epoxy resin, and polished using diamond abrasive materials. The studies were conducted using a MIM-8M model metallographic microscope. The results of the petrographic studies are presented in Figure 4 and Table 5.

The petrographic analysis of the samples showed that the dust structure consists mainly of spinel, forsterite, and glass. Small amounts of magnesium and calcium aluminosilicates were found in the intermediate phase.

Table 5 presents the phase composition of these samples based on the results of visual counting in polished sections.

From Figure 4, it is evident that the crystallization pattern in the materials is quite homogeneous. Among the main mass of forsterite, a significant amount of the glass phase is present. The forsterite crystals exhibit microcracks and a resorbed surface. Spinelid is present in smaller quantities. Its crystals are smaller than those of forsterite and have a high reflectivity.

## 3. Results and Discussion

The examined dust samples are characterized by uneven microporosity, ranging from 5% to 15%. The presence of the identified minerals (Table 5) in the analyzed samples is confirmed by the observations in immersion liquids and X-ray phase analysis.

Thus, based on the phase composition, the examined dust from dry gas cleaning systems represents a mixture of forsterite (up to 30 vol.%), spinel (up to 15 vol.%), and a complex glassy phase (up to 40 vol.%).

Taking into account the obtained data on the physicochemical characteristics of the fine fraction (AD) and the filler material, laboratory experiments were conducted to agglomerate them using the briquetting method. Briquetting was carried out with two types of mixtures: in the first case, only AD was briquetted, and in the second case, a mixture of AD and dry gas cleaning dust (referred to as DGD) was used. A polymer-based binder (hereafter referred to as Polymer) served as the binding agent. The compositions of the briquetted materials are presented in Table 6 below.

After dosing the charge components according to Table 6, dry mixing was carried out in an EIRICH EL1 mixer until the mixture was fully homogenized. The resulting mixture was then moistened and further mixed until a uniform consistency was achieved. The moisture content ranged from 3% to 5% of the dry material’s mass. The prepared mixture was loaded into a mold (channel diameter—30 mm) and briquetted using an IP-1000-1 press with a compression force of 30 kN per briquette (equivalent to 425 kg per 1 cm^2^).

The resulting briquettes were subjected to forced drying at 120 °C for 3 h.

After drying, the strength of the briquettes was measured in terms of splitting resistance. The results of the strength measurements for the briquettes, categorized by mixture variants, are presented in Table 7.

**Figure 5:**
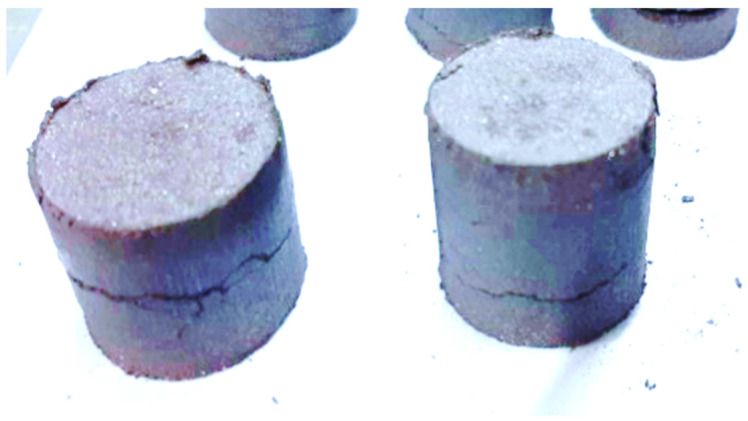
Appearance of the Briquettes.

It is worth noting that briquetting mixtures composed solely of fine fraction (AD) (variants No. 1–2), regardless of the binder consumption, exhibited extremely low green strength and easily disintegrated. Increasing the compression force resulted in the formation of deep transverse cracks and ruptures, indicating over-pressing. This phenomenon is visually demonstrated in Figure 5.

**Figure 6:**
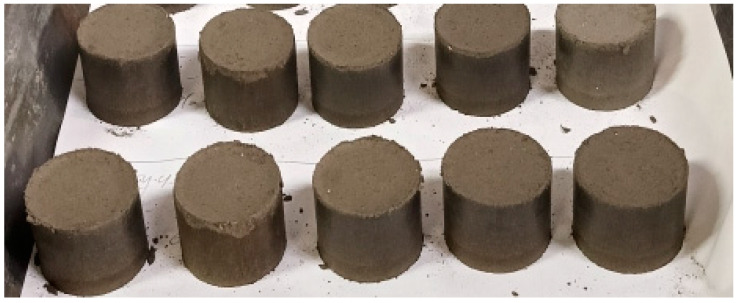
Appearance of Briquettes Made from the AD and DGD Mixture.

The introduction of inert fillers in the form of DGD had a positive effect (mixtures No. 3–4). The briquettes were dense, free of transverse cracks, and exhibited sufficient green strength. The appearance of these briquettes is shown in Figure 6.

According to Table 7, the briquettes made with filler material exhibit high mechanical strength in terms of splitting resistance. The green and dried briquettes’ strengths range from 34 to 41 kg/briquette and 297 to 505 kg/briquette, respectively, compared to the required values of 20 kg/briquette and 150 kg/briquette for briquetted chrome-containing raw materials.

In addition to the strength characteristics of dry briquettes, the hot strength of the agglomerated raw material is of particular significance. This characteristic indicates the behavior of the briquettes under thermal stress, which they experience during the smelting process in furnaces. The lower the hot strength, the higher the risk of premature briquette failure, leading to dust emissions or disruption of the smelting process.

To assess the hot strength of the developed briquette formulation, additional tests were conducted. For this, using the mixture compositions from Table 7, 10 briquettes were made for each variant. The briquettes were made to the same height with flat ends using a mold with a channel diameter of 20 mm.

The prepared briquettes were placed in a Nabertherm TR420 drying oven and dried for 2 h at 120 °C (starting from the temperature rise). After drying, the briquettes (in a vertical position) were placed in a Nabertherm N7/H muffle furnace preheated to 1050 °C. A steel load of a rectangular cross-section (referred to as the load) was carefully placed on top of the briquettes. The load on each briquette was calculated to be 0.52 kg/cm^2^, so the load on one briquette was 1.63 kg, and on two briquettes, 3.26 kg.

**Figure 7:**
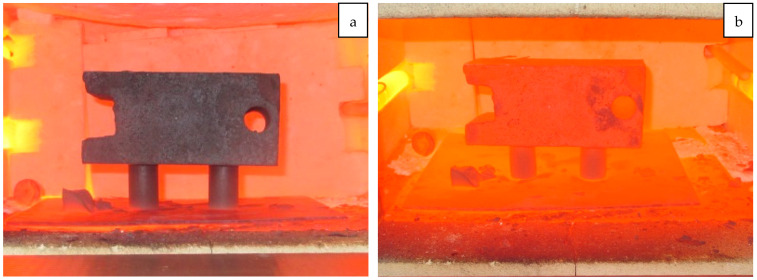
Testing of Briquettes for Hot Strength Under Load. (**a**) At the initial stage; (**b**) after 60 min.

The briquettes were held at the specified temperature for 60 min. Every 5 min during the thermal treatment, the furnace lid was opened to observe the condition of the briquettes (Figure 7).

**Figure 8:**
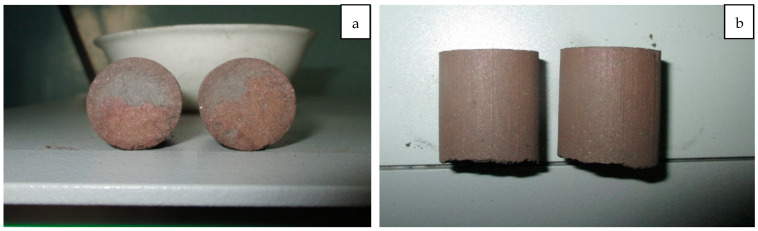
Condition of Briquettes After Thermal Treatment Under Load ((**a**)—side view, (**b**)—top view).

After the holding time, the load was carefully removed, and the briquettes were extracted and inspected for deformation (shrinkage) and cracks (Figure 8).

### Subsection

The visual inspection of the briquettes did not reveal any cracks throughout their height, including the end faces. Briquettes from both variants also retained their original height, indicating their high resistance to thermal deformation under load at temperatures up to 1050 °C. This temperature corresponds to the temperature range of the upper layers of the charge in ferroalloy furnaces.

To model the behavior of the briquettes in the middle and lower layers of the charge (in the temperature range of 1100–1600 °C), further experiments were conducted to determine the degree of softening (shrinkage) of the experimental briquettes.

**Figure 9:**
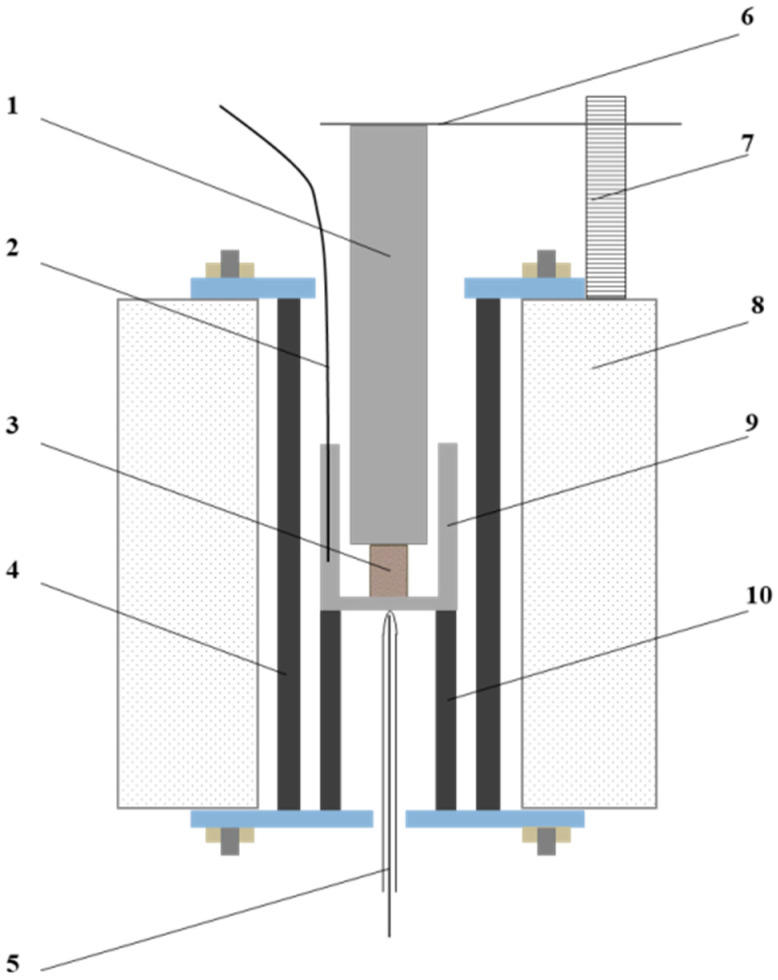
Schematic of the Laboratory Setup. 1—load; 2—upper thermocouple; 3—test briquette; 4—graphite heater; 5—lower thermocouple; 6—level gauge; 7—ruler; 8—furnace body; 9—refractory crucible; and 10—stand.

The experiments were conducted in a high-temperature Tammann furnace (schematic in Figure 9), where a refractory crucible with a briquette was loaded. A special cylindrical refractory weight was placed on top of the briquette.

The internal diameter of the crucible is slightly larger than the diameter of the load pressing on the briquette. For more precise temperature control during the experiment, a slit was made inside the crucible, where a thermocouple was installed, ensuring that the thermocouple does not interfere with the free movement of the load. At the initial stage, the end of the thermocouple is positioned at the middle of the briquette’s height. The briquette was heated at a rate of 6–8 °C per minute from room temperature, with temperature being recorded at 1 mm shrinkage intervals.

**Figure 10:**
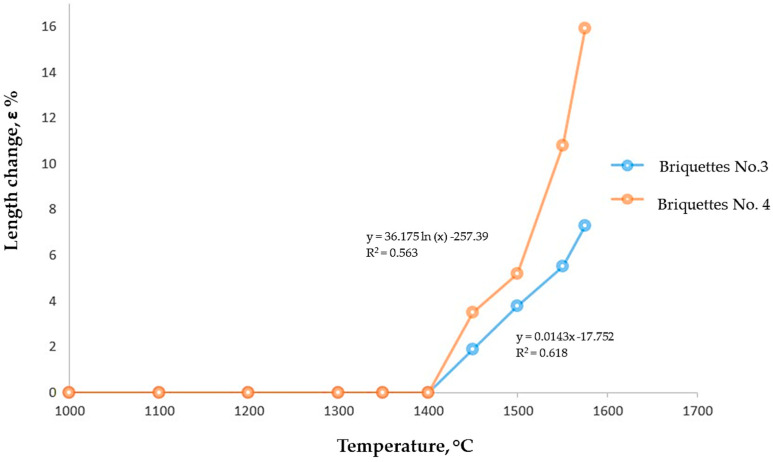
Dependence of Softening Degree on Temperature.

The results of the tests to determine the thermoplastic characteristics of the briquettes are presented as a dependence of softening degree (shrinkage) on temperature in Figure 10.

Up to a temperature of 1400 °C, no changes were observed. After that, a gradual shrinkage of the briquettes was noted with increasing temperature. For briquette No. 3, when the maximum temperature in the crucible reached 1550 °C, the shrinkage level was 8%, while for briquette No. 4, the shrinkage reached 16%. A further temperature increase to 1600 °C led to the partial melting of the briquettes.

It is possible that the exceptionally high thermoplastic properties of the briquettes are related to the presence of PSG, which contains refractory phases such as forsterite and spinel. These phases serve as a kind of refractory framework for the briquettes. Typically, the initial signs of melting for pure AD occur within the temperature range of 1400–1450 °C. In our case, this range shifts to 1550 °C, ensuring the sufficient gas permeability of the charge column at temperatures close to melting. However, these findings require further investigation on an enlarged laboratory scale under conditions as close as possible to industrial operations.

**Figure 11:**
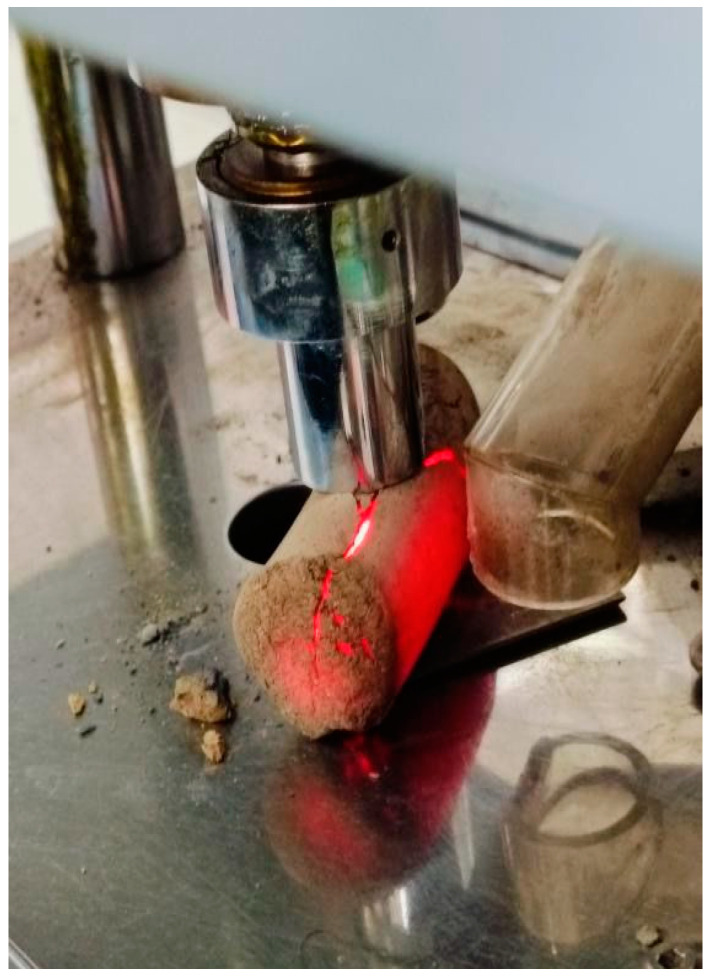
Measurement of Hot Splitting Strength of Briquettes.

The next batch of briquettes was also heated to 1050 °C, but without the load. After 20 min of heating, the briquettes were carefully removed from the furnace using special tongs and placed (while still hot) into the RB-1000 testing press to measure the hot splitting strength (Figure 11). The strength measurement results are presented below in Table 8.

The data in Table 8 show that both briquette variants exhibit a significantly higher hot splitting strength than the required parameters.

Next, to evaluate the influence of DGD and the binder on the chemical composition of the final product, laboratory experiments were conducted to smelt high-carbon ferrochrome from the two aforementioned briquette variants.

The experiments were conducted in a Nabertherm LHT 08/17 indirect heating high-temperature furnace. The briquette charge weight was 400 g for each variant. The briquettes were weighed and placed in an alumina crucible. The crucible with the material was then placed in the furnace and heated to 1720 °C. Once the liquid melt was observed, the sample was held at this temperature for 20 min. The total duration of one experiment, including temperature ramp-up and holding time, was 3 h.

After the holding time, the furnace power was turned off, and after 2–2.5 h of cooling, the crucible with the smelted products was removed and separated, with the ingot, slag, and crucible material being separated.

**Figure 12:**
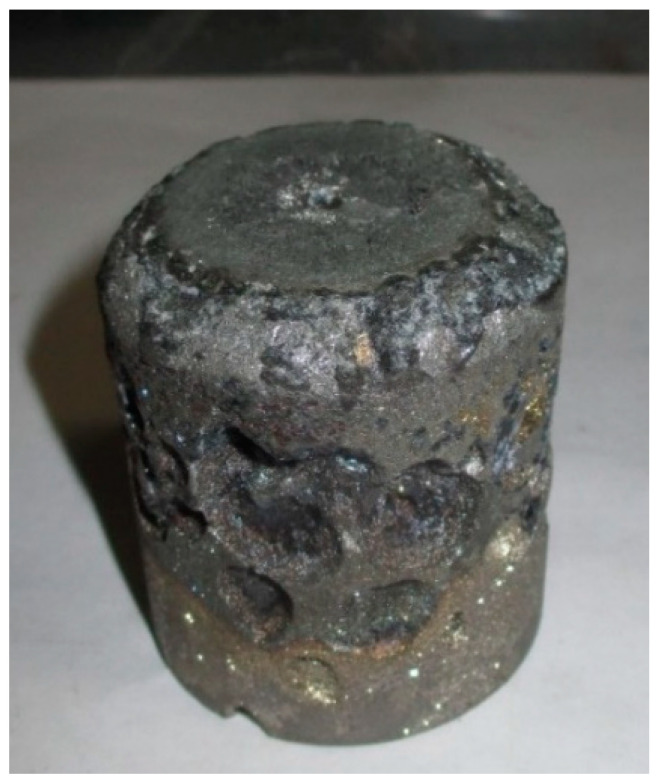
Metal Ingot Smelted from Briquettes.

**Figure 13:**
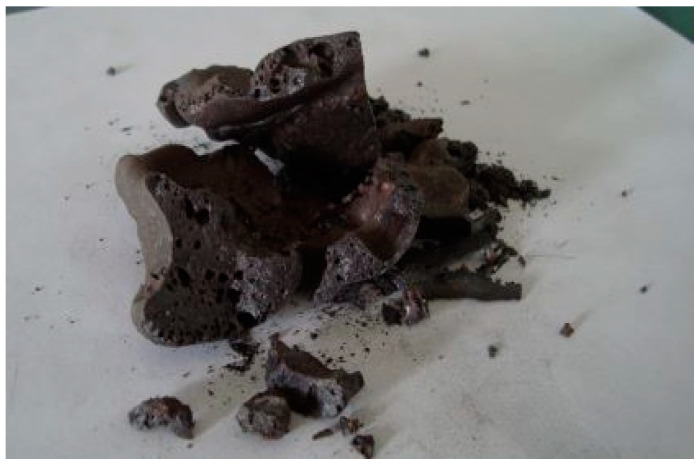
Slag Sample from Briquette Smelting Experiments.

Figure 12 and Figure 13 show images of the metal ingots and slag obtained from the agglomerated material. These images reveal that, during the experiments, the briquettes fused into a solid ingot.

The metal ingots were clean, with no traces of slag or crucible material adhering to their surface. The fracture across the entire height of the ingot revealed homogeneous metal, with no signs of unmelted material. The ingot structure was dense, without distinct pores or blowholes typical for HC FeCr.

Table 9 presents the data on the mass of the initial and final products of the performed smelting experiments.

The obtained metal corresponded in chemical composition to high-carbon ferrochrome grade HC FeCr800. Table 10 and Table 11 present the chemical compositions of the metal in comparison with standard HC FeCr (according to ISO 5448 [23]) and the slag (compared to industrial samples).

Typically, in the crushing sections of high-carbon ferrochrome (HCFeCr), the aspirated dust is a mixture of dust from the crushing of various grades—FeCr800, FeCr850, and FeCr900. The production of FeCr800 alloy during remelting may be explained by the increased oxidation of fine particulate material, which led to a reduction in the carbon concentration in the metal due to the release of CO from the carbides during remelting.

On the other hand, the reduction in the carbon content in the final alloy may be caused by the refining process, where carbon is removed from the raw material (AD) by Cr_2_O_3_, SiO_2_, and FeO oxides present in the DGD composition.

The chemical composition of the slags obtained during experimental smelting does not differ significantly from the classical slags formed during the production of HC FeCr in industrial conditions.

The experiments on smelting HC FeCr from briquetted material allowed for the production of ferrochrome that meets the requirements for finished products according to the standard [23] used at domestic enterprises. The levels of harmful impurities, such as S and P, are below the allowable limits. The chromium oxide content is also below the range of Cr_2_O_3_ content found in industrial slags.

The slag multiplicity is in the range of 0,01–0,02 compared to 0,5 in the classical remelting of AD in bulk with slag addition. That is, the use of briquettes made from a mixture of AD and PSG, when potentially combined with technologies, will not lead to an increase in slag multiplicity and, consequently, the loss of the main element in the form of metal droplets with slag.

Overall, the briquetting technology for AD tested on a laboratory scale, with the inclusion of PSG dust in the mixture and the use of polymer-based binder, based on the study of the physicochemical properties of these materials, demonstrated the feasibility of producing standard grades of ferrochrome. The addition of PSG dust to AD in a 4:1 ratio, with a polymer binder consumption of 3% (of the dry mixture weight), allows the production of briquettes with high-strength characteristics sufficient for further metallurgical processing.

## 4. Conclusions

The analysis of the physicochemical characteristics of AD (aspirated dust) shows that the material is sufficiently dispersed and its chemical composition is almost identical to that of standard grades of high-carbon ferrochrome. The phase composition is mainly represented by Cr_7_C_3_ carbide and free carbon in the form of graphite.

Agglomerating AD in its pure form through briquetting is not feasible due to the specific grain structure and low wettability. The use of filler material in the form of dust from the baghouse filters of dry gas cleaning systems in furnaces producing high-carbon ferrochrome enhances the interparticle adhesion of AD, ultimately allowing for its agglomeration by briquetting.

In addition, the baghouse dust acts as an additional source of chromium and a slag-forming component, which will help exclude the use of recycle slag during the remelting of AD.

Briquetting the mixture of AD and baghouse dust with polymer-based binders results in briquettes whose splitting strength exceeds the requirements for agglomerated raw materials for ferrochrome production by 2–3 times.

The hot splitting strength indicators of the briquettes are higher than the required parameters for ferroalloy processes. The inclusion of baghouse dust in the briquette composition improves their thermoplastic properties. Briquette shrinkage occurs at higher temperatures, thanks to the refractory properties of baghouse dust (1590 °C), which, due to the integrity of the briquettes, will ensure better gas dynamics in the high-carbon ferrochrome smelting process.

The remelting of experimental briquettes demonstrated the feasibility of producing standard ferrochrome grades that fully meet consumer requirements in terms of chemical composition.

## Figures and Tables

**Table 1:** Chemical Composition of Aspiration Dust (AD), %.

Cr	Si	C	S	P	Fe
64.89	2.54	8.24	0.027	0.014	other

**Table 2:** Particle Size Distribution of Aspiration Dust (AD).

Size Class, mm	+0.2	+0.16	+0.125	+0.071	−0.071
As a percentage, %	0.07	0.14	0.31	2.75	96.65

**Table 3:** Chemical Composition of Dust from Bag Filters, %.

MgO	SiO_2_	CaO	Cr_total_	Al_2_O_3_	C
32.8	18.0	0.4	14.9	4.2	5.8

**Table 4:** Particle Size Distribution of Aspiration Dust (AD).

Size Class, mm	+0.1	+0.071	+0.04	+0.02	−0.02
As a percentage, %	1.02	1.84	3.35	6.65	87.14

**Table 5:** Phase Composition of the Studied Materials.

Phase Type	Phase Content, Volume, %
Forsterite (Ng = 1.670, Np = 1/635)	20–30
Spinelid:	
Colorless N = 1.726	10–15
Pink N = 1.750	-
Glass, N = 1.600	30–40
Ore body	up to 2
Aluminosilicates	up to 4
The metal phase	up to 4

**Table 6:** Compositions of Mixtures for Briquetting.

Variants	Material	Binding Agent	Expenditure, % *
1	AD	Polymer	3
2	AD	Polymer	4
3	AD + DGD (the ratio is 80/20)	Polymer	3
4	AD + DGD (the ratio is 80/20)	Polymer	4

*—consumption of polymer binder.

**Table 7:** Results of the split strength of briquettes.

Variant	Composition	Binding, %	W, %	Splitting Strength, kg/Briquette	
				Raw	Drying at 120 °C for 3 h
3	AD + DGD (80:20)	3	5	34	297
4	AD + DGD (80:20)	4	5	41	505

**Table 8:** Hot Splitting Strength Indicators.

Variant	Splitting Strength, kg/Briquette
3	54.5
4	60.5
Required	At least 40.0

**Table 9:** Masses of Initial and Final Smelting Products.

No.	Name	Briquettes No. 3	Briquettes No. 4
1	Crucible mass, g	241.03	242.65
2	Mass of the melt with crucible after smelting, g	635.4	633.58
3	Losses during calcination, g/%	5.63/1.41	9.07/2.27
4	Mass of the obtained ingot, g	384.79	385.47
5	Mass of slag, g/%	9.58/2.49	5.46/1.42

**Table 10:** Chemical Composition of the Obtained Metal, %.

Material	Cr	C	S	P
Briquettes No. 3	68.4	7.72	0.022	0.016
Briquettes No. 4	68.8	7.64	0.023	0.016
HC FeCr (ISO 5448)	Not less than 65.0	Not less than 8.0	Not less than 0.03	Not less than 0.06

**Table 11:** Chemical Composition of the Obtained Slag, %.

Material	Cr_2_O_3_	SiO_2_	CaO	MgO	Al_2_O_3_	FeO
Briquettes No. 3	2.61	27.17	2.88	51.19	15.46	0.66
Briquettes No. 4	2.64	27.45	2.21	51.32	15.68	0.67
Slag HC FeCr	3–5	27.0–30.5	2.0–3.2	45–52	15–19	0.6–0.8

## Data Availability

The original contributions presented in this study are included in the article. Further inquiries can be directed to the corresponding author(s).
